# Complete genome sequences of seven *Ralstonia solanacearum* phylotype IIB-4C/IIB-4E strains isolated from plantain trees in Ecuador

**DOI:** 10.1128/mra.00517-25

**Published:** 2026-02-23

**Authors:** Rommel Guevara, Teresa Guerrero, Nicol Barriga, Erika Muñoz, Denisse Benítez, Paúl Cárdenas

**Affiliations:** 1 Instituto de Microbiología, Universidad San Francisco de Quito 27902, Quito, Pichincha, Ecuador; 2 Biosiembra, EcoHarmony S.A., Quito, Pichincha, Ecuador; University of Strathclyde, Glasgow, United Kingdom

**Keywords:** *Ralstonia solanacearum*, moko disease, plantain disease

## Abstract

*Ralstonia solanacearum* has been causing an epidemic of Moko disease in Ecuador since early 2024. Here, we announce the publication of seven complete genome sequences from isolates belonging to the phylotype IIB sequevar IIB-4 and subclade IIB-4C/IIB-4E. Their genome megaplasmid is predicted to contain a class D beta-lactamase gene.

## ANNOUNCEMENT

Ecuador faces an epidemic of Moko disease (1) since early 2024. We provide complete genomes of seven isolates from infected trees or surrounding soil.

We collected pseudostem tissue or surrounding soil between February and May 2024 (Table 1). A piece of tissue from the wounded area was retrieved, cleaned with water, surface-sterilized with 70% ethanol, cut with a scalpel, and gentle pressure applied to obtain the bacterial exudate for inoculation (2). Ten grams of soil (50 cm away and 15 cm deep) was mixed with sterile water (1:10), supernatant retrieved and diluted to 10^−4^ prior to inoculation (2). Processed samples were inoculated in SMSA plates and incubated for 72 h at 28°C aerobically (3). Presumptive *Ralstonia* colonies were isolated for genomic DNA extraction by restreaking in NASYE medium and incubated for 48–72 h at 28°C under aerobic conditions (4). DNA was extracted using the ZymoBIOMICS DNA Microprep Kit and quantified with Qubit 1X dsDNA HS assay. Libraries were prepared with the Native Barcoding Kit 24 V14 (ONT), without fragmentation or size selection, and sequenced in the GridION (R10.4.1 flow cells) with super-accurate basecalling (MinKNOW v23.11.7). Sequencing yielded 1.30M of demultiplexed raw reads (4.03 Kb N50 and 21 Q-score). Genomes were assembled with Flye v2.9.5 (5) and Raven v1.8.3 (6), processed with Trycycler v0.5.5 (7), and polished with Medaka v1.12.1. Default parameters were used for all software. Closed circular assemblies were returned by Trycycler for the three replicons in all isolates. Replicons were not reoriented. Completeness was assessed with Busco v5.8.2 (8) (data set: burkholderiales_odb10) and Quast v5.2.0 (9) (reference: NZ_CP012943.1). Taxonomy assignment and annotation were performed by NCBI with PGAP v6.8 (10). Phylogeny was inferred following the KBase narrative proposed previously (11), using the tool Insert Set of Genomes Into SpeciesTree v2.2.0 with the reference genome set RSSC_for_phylogenomics_v2023-11-11.

**TABLE 1:** Characteristics of isolates sequenced in this study^*a*^

Isolate	Location	Date	SRA accession	Raw read number	Raw read N50 (bp)	NCBI accession	Size (bp)	ANI value (%)
							Chromosome	
							Plasmid 1	
							Plasmid 2	
068_2	Buena Fe, Los Ríos	08 February 2024	SRR33189762	80,886	6,323	ASM3969333v1	3503462	96.14
							1951654	
							173870	
077-080_1	El Empalme, Los Ríos	08 February 2024	SRR33189761	641,114	1,550	ASM3969254v1	3503477	96.14
							1951655	
							173870	
095-3	San Jacinto del Búa, Santo Domingo	20 May 2024	SRR33189767	190,622	1,986	ASM4171472v1	3500663	96.15
							1941694	
							174509	
102-3	San Jacinto del Búa, Santo Domingo	20 May 2024	SRR33189766	59,593	2,958	ASM4171471v1	3500655	96.15
							1941693	
							174509	
071-2	Arenillas, El Oro	20 May 2024	SRR33189765	82,526	2,693	ASM4171470v1	3500695	96.14
							1950768	
							173863	
144-1	La Unión, Los Ríos	20 May 2024	SRR33189764	128,087	4,544	ASM4171469v1	3500665	96.15
							1944777	
							172529	
115-4	Arenillas, El Oro	20 May 2024	SRR33189763	122,762	5,486	ASM4171468v1	3500755	96.14
							1950766	
							173863	

^*a*^ All isolates were retrieved from pseudostems, except isolate 102-3 collected from soil. Only isolates 095-3 and 102-3 came from the same farm.

**Fig 1:**
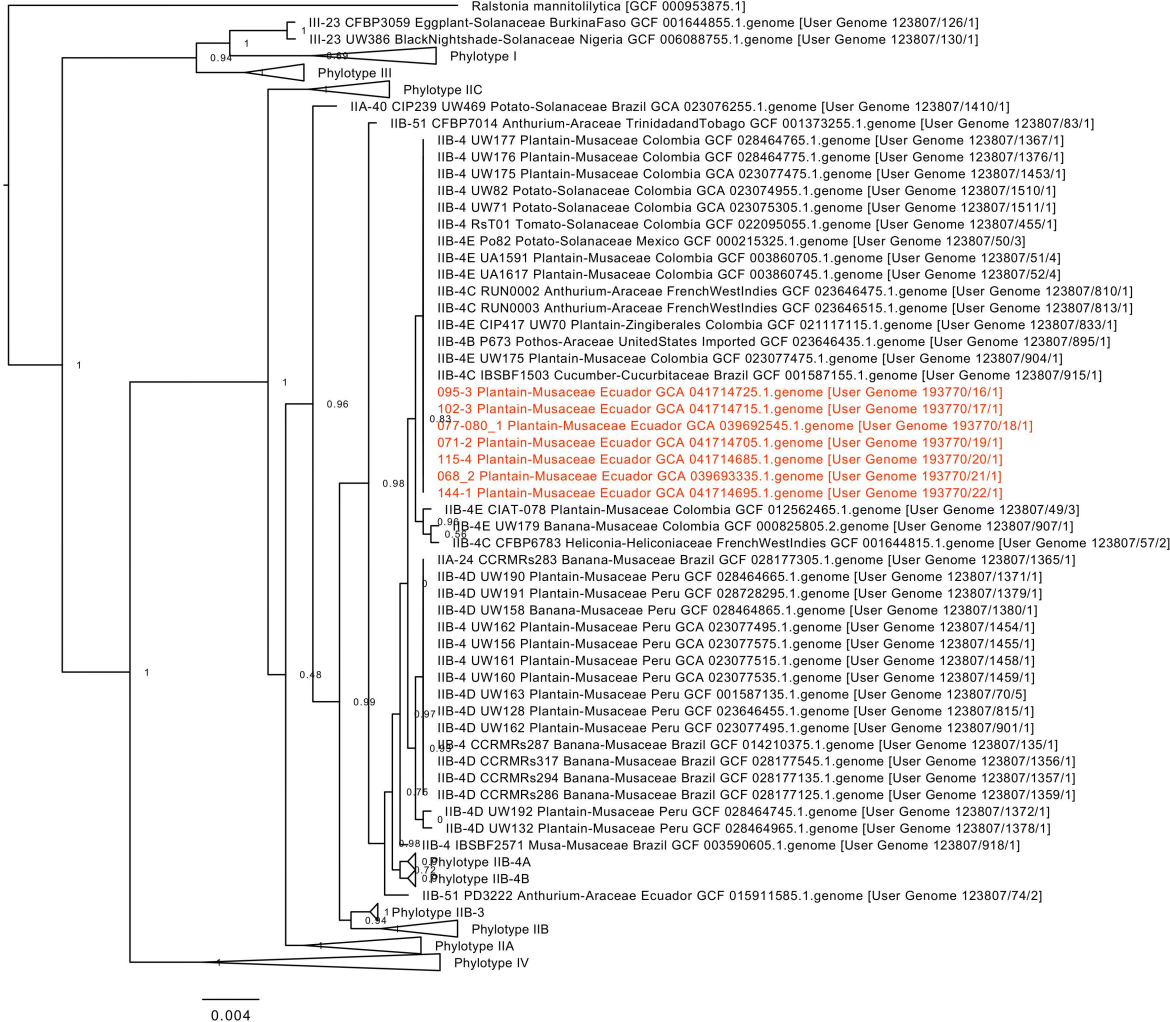
Phylogeny of isolates sequenced in this study (shown in red). Node labels are local support values computed with the KBase tool.

Seven closed circular genomes were obtained (66.5% GC content for all), each with a chromosome and two plasmids. All isolates were confirmed as *Ralstonia solanacearum* (ANI values to GCA_015910705.1 in Table 1), and all scored 99% BUSCO completeness and 88% Quast similarity. Genome annotation identified a predicted class D beta-lactamase gene in the megaplasmid of each isolate. Phylogeny clustered all strains within phylotype IIB-4C/IIB-4E (Fig. 1).

## Data Availability

Genome sequences and raw read data are deposited in NCBI (accession numbers in Table 1).

## References

[1] Drenth A, Kema G. 2021. The vulnerability of bananas to globally emerging disease threats. Phytopathology 111:2146–2161. doi:10.1094/PHYTO-07-20-0311-RVW 34231377

[2] Obrador-Sánchez JA, Tzec-Simá M, Canto-Canché B, Higuera-Ciapara I. 2017. Técnicas para el aislamiento, identificación y caracterización molecular de cepas de Ralstonia solanacearum relacionadas a Moko del plátano. RMF 35. doi:10.18781/R.MEX.FIT.1705-1

[3] Popoola A, Ganiyu S, Enikuomehin O, Bodunde J, Adedibu O, Durosomo H, Karunwi O. 2015. Isolation and characterization of Ralstonia solanacearum causing bacterial wilt of tomato in Nigeria. Nig J Biotechnol 29:1. doi:10.4314/njb.v29i1.1

[4] Gill JJ, Svircev AM, Smith R, Castle AJ. 2003. Bacteriophages of Erwinia amylovora. Appl Environ Microbiol 69:2133–2138. doi:10.1128/AEM.69.4.2133-2138.2003 12676693 PMC154828

[5] Kolmogorov M, Yuan J, Lin Y, Pevzner PA. 2019. Assembly of long, error-prone reads using repeat graphs. Nat Biotechnol 37:540–546. doi:10.1038/s41587-019-0072-8 30936562

[6] Vaser R, Šikić M. 2021. Time- and memory-efficient genome assembly with Raven. Nat Comput Sci 1:332–336. doi:10.1038/s43588-021-00073-4 38217213

[7] Wick RR, Judd LM, Cerdeira LT, Hawkey J, Méric G, Vezina B, Wyres KL, Holt KE. 2021. Trycycler: consensus long-read assemblies for bacterial genomes. Genome Biol 22:266. doi:10.1186/s13059-021-02483-z 34521459 PMC8442456

[8] Simão FA, Waterhouse RM, Ioannidis P, Kriventseva EV, Zdobnov EM. 2015. BUSCO: assessing genome assembly and annotation completeness with single-copy orthologs. Bioinformatics 31:3210–3212. doi:10.1093/bioinformatics/btv351 26059717

[9] Gurevich A, Saveliev V, Vyahhi N, Tesler G. 2013. QUAST: quality assessment tool for genome assemblies. Bioinformatics 29:1072–1075. doi:10.1093/bioinformatics/btt086 23422339 PMC3624806

[10] Tatusova T, DiCuccio M, Badretdin A, Chetvernin V, Nawrocki EP, Zaslavsky L, Lomsadze A, Pruitt KD, Borodovsky M, Ostell J. 2016. NCBI prokaryotic genome annotation pipeline. Nucleic Acids Res 44:6614–6624. doi:10.1093/nar/gkw569 27342282 PMC5001611

[11] Lowe-Power TM, Avalos J, Bai Y, Munoz MC, Chipman K, Elmgreen VN, Prasad N, Ramirez B, Sandhar A, Tom CE, Williams D. 2020. A Meta-analysis of the known global distribution and host range of the Ralstonia species complex. biorxiv. doi:10.1101/2020.07.13.189936

